# Human β-defensin 3 gene modification promotes the osteogenic differentiation of human periodontal ligament cells and bone repair in periodontitis

**DOI:** 10.1038/s41368-020-0078-6

**Published:** 2020-04-29

**Authors:** Lingjun Li, Han Jiang, Rixin Chen, Jing Zhou, Yin Xiao, Yangheng Zhang, Fuhua Yan

**Affiliations:** 1 Nanjing Stomatological Hospital, Medical School of Nanjing University, Nanjing, China; 2 People’s Hospital of Suzhou National New & Hi-Tech Industrial Development Zone, Suzhou, China; 3 The Affiliated Stomatological Hospital, Zhejiang University School of Medicine; Key Laboratory of Oral Biomedical Research of Zhejiang Province, Zhejiang University School of Stomatology, Hangzhou, China; 4 Institute of Health and Biomedical Innovation & the Australia-China Centre for Tissue Engineering and Regenerative Medicine, Queensland University of Technology, Brisbane, Australia

**Keywords:** Periodontitis, Stem-cell research

## Abstract

Efforts to control inflammation and achieve better tissue repair in the treatment of periodontitis have been ongoing for years. Human β-defensin 3, a broad-spectrum antimicrobial peptide has been proven to have a variety of biological functions in periodontitis; however, relatively few reports have addressed the effects of human periodontal ligament cells (hPDLCs) on osteogenic differentiation. In this study, we evaluated the osteogenic effects of hPDLCs with an adenoviral vector encoding human β-defensin 3 in an inflammatory microenvironment. Then human β-defensin 3 gene-modified rat periodontal ligament cells were transplanted into rats with experimental periodontitis to observe their effects on periodontal bone repair. We found that the human β-defensin 3 gene-modified hPDLCs presented with high levels of osteogenesis-related gene expression and calcium deposition. Furthermore, the p38 MAPK pathway was activated in this process. In vivo, human β-defensin 3 gene-transfected rat PDLCs promoted bone repair in SD rats with periodontitis, and the p38 mitogen-activated protein kinase (MAPK) pathway might also have been involved. These findings demonstrate that human β-defensin 3 accelerates osteogenesis and that human β-defensin 3 gene modification may offer a potential approach to promote bone repair in patients with periodontitis.

## Introduction

Periodontitis, a chronic inflammatory disease induced by dental plaque, damages the integrity of tooth-supporting tissues.^1^ It can disturb normal bone metabolism and eventually result in alveolar bone loss.^2^ According to epidemiological investigations, at least one-half of the world’s population suffers from periodontitis, and it has become the eleventh disease among global diseases that cause short- or long-term loss of health.^3^ Poor periodontal condition is a major problem that affects the oral health of people in China.^4^ Inflammatory responses and bone loss due to periodontitis have become the most critical and challenging problems to be solved for individuals to achieve healthy periodontium.^5,6^

Human β-defensin 3 (hBD3), a small molecule cationic antimicrobial peptide consisting of 45 amino acids, is thought to be among the most promising antimicrobial peptides because of its broad-spectrum antibiotic activity.^7,8^ In addition, hBD3 exhibits diverse functions in host defences, including in immune regulation and inflammatory processes.^9,10^ It was reported that hBD3 has significant anti-inflammatory activity in the host by regulating Toll-like receptor signalling pathways.^11^ Kiatsurayanon et al.^12^ found that hBD3 could regulate the innate immunity of skin by increasing the expression of several claudins, inducing claudin localization along cell–cell borders and reducing the paracellular permeability of keratinocyte layers. Moreover, hBD3 also plays a pivotal role in cell proliferation and differentiation. hBD3 potentially promotes the proliferation of periodontal ligament (PDL) fibroblasts^13^ and the osteogenic differentiation of osteoblast-like human osteosarcoma cells (MG63 cells).^14^

The application of hBD3 in periodontitis treatment has been studied for years.^15,16^ Bedran et al.^17^ showed that hBD3 had anti-inflammatory activity in a three-dimensional (3D) coculture model of gingival epithelial cells and fibroblasts. Our team previously demonstrated that recombinant hBD3 inhibits periodontitis development by suppressing inflammatory responses in macrophages and modulates macrophage activation during the acute inflammatory response to *Porphyromonas gingivalis* lipopolysaccharides (LPS).^18,19^ In an in vivo study, transplantation of periodontal ligament cell (PDLC) sheets expressing hBD3 promoted bone repair and osteocalcin (OCN) expression in periodontal tissues.^20^ In addition to the its antimicrobial, anti-inflammatory and immune regulation effects, hBD3 affects cell differentiation during these processes. PDLCs derived from the PDL possess stem cell-like attributes and have excellent potential for periodontal regeneration.^21,22^ Thus it is reasonable to hypothesize that increasing hBD3 expression in PDLCs may contribute to periodontal regeneration.

Genetic engineering technology has been widely used to transfer relevant exogenous genes into a host to produce valuable proteins or peptides, such as hBD3. By gene transfection, we can achieve more stable and sustained expression of target proteins.^23^ Our team has successfully modified the hBD3 gene and produced a stable level of hBD3 in hBD3-engineered human PDLCs (hPDLCs). Hence, we investigated whether this hBD3 gene modification promotes the osteogenic differentiation of hPDLCs.

The process of osteogenic differentiation was proven to be modulated by various signalling pathways.^24,25^ Mitogen-activated protein kinases (MAPKs) act as prominent intracellular enzymes and can be activated by extracellular stimuli, enabling cells to participate in various activities, including apoptosis, neutrophil-mediated inflammatory processes, wound healing and tissue remodelling.^26^ Among all the MAPK pathways, the p38 MAPK pathways seems to be the most closely related to osteogenesis. Lee et al.^27^ reported that osteoblast differentiation could be enhanced by berberine through the p38 MAPK-Runx2 pathway both in vitro and in vivo. Furthermore, it was proven that the p38 MAPK pathway also modulates the proliferation and osteogenic differentiation of human bone-derived marrow mesenchymal stem cells.^28^ There are few reports about hBD3 and osteogenesis; however, other cationic antimicrobial peptides, such as LL37, have been found to affect the proliferation and differentiation of MC3T3-E1 cells^29^ and to enhance bone regeneration in a rat calvarial bone defect through the p38 MAPK pathway.^30^ Therefore, we hypothesized that the p38 MAPK pathway might also modulate the osteogenic process of hBD3 gene-modified hPDLCs.

In this study, a recombinant adenovirus vector carrying the hBD3 gene was successfully constructed. Then the osteogenic differentiation of hPDLCs with hBD3 gene modification in an inflammatory environment and the potential mechanisms were investigated. Further experiments were conducted in vivo to observe the effects of rat PDLCs (rPDLCs) with hBD3 gene modification on periodontal repair and regeneration in the rat periodontitis model.

## Results

### Infection efficiency of Ad-hBD3 and hBD3 overexpression in the hPDLCs

**Fig. 1:**
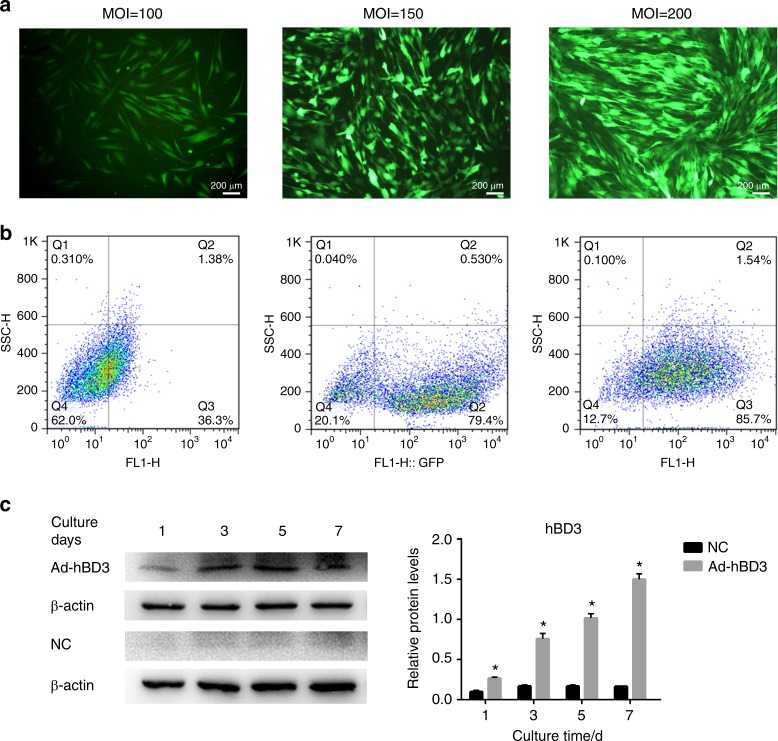
Ad-hBD3 transfection and overexpression of hBD3 in the hPDLCs. **a** Fluorescence images of the hPDLCs transfected with Ad-hBD3 transfected at different MOI values ranging from 100 to 200. **b** Flow cytometric analysis of the transfected hPDLCs transfected at different MOI values ranging from 100 to 200. **c** The hBD3 protein expression level of the transfected hPDLCs on days 1, 3, 5, and 7 at an MOI of 150 (*P < 0.05, where asterisk (*) indicates a significant difference compared with the NC group)

A positive correlation between the multiplicity of infection (MOI) value and mean fluorescence intensity was observed with adenoviral MOI values ranging from 100 to 200 (Fig. 1a). The flow cytometric results (Fig. 1b) showed the same tendency, with hPDLCs transfected at 150 and 200 MOI displaying high transfection efficiency. However, the death rate of the hPDLCs increased at 200 MOI. Hence, MOI 150 was chosen for the following experiments. hPDLCs transfected with Ad-hBD3 showed high hBD3 gene and protein expression levels (Fig. 1c) from day 3 to day 7.

### Effects of hBD3 gene transfection on the osteogenic differentiation of the hPDLCs

**Fig. 2:**
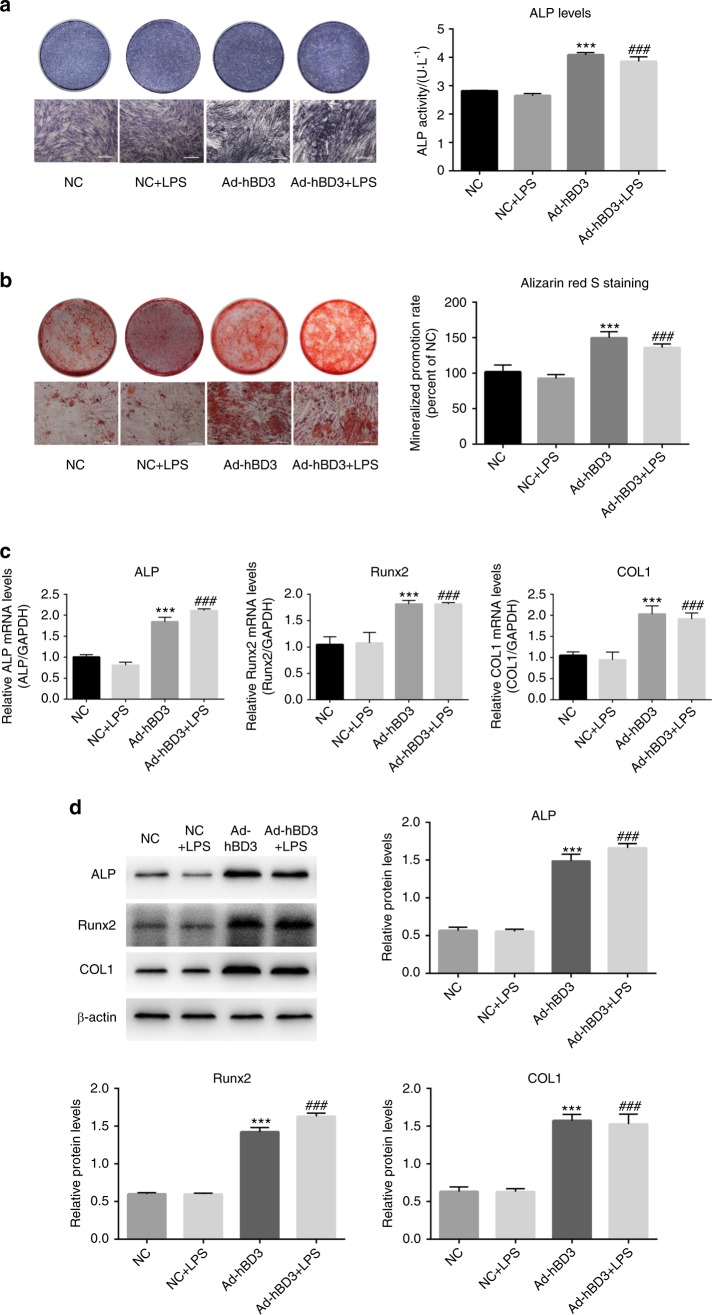
Effects of Ad-hBD3 gene transfection on the osteogenic differentiation of the hPDLCs in an inflammatory environment. hPDLCs were transfected with Ad-hBD3 or NC (Ad-GFP) and treated with or without *E. coli* LPS. **a** ALP staining and ALP activity on day 7. **b** Alizarin red S staining of the mineralized nodules on day 21. **c** qRT-PCR and **d** western blot analysis of ALP, Runx2, and COL1 expression on day 7. (***, ^###^*P* < 0.001, where triple asterisks (***) indicate a significant difference compared with the NC group, and triple hashes (###) indicate a significant difference compared with the NC+LPS group)

*Escherichia coli* LPS (1 μg·mL^−1^) was added to cells to stimulate an inflammatory microenvironment.^31^ alkaline phosphatase (ALP) and alizarin red S (ARS) staining assays were conducted to detect any osteogenic differentiation changes. Quantitative real-time PCR (qRT-PCR) and western blotting (WB) were performed to observe the osteogenesis-related gene and protein expression in hPDLCs. The results demonstrated that ALP and ARS staining were darker in both the Ad-hBD3 and Ad-hBD3+LPS groups (Fig. 2a, b). The mRNA and protein expression levels of ALP, Runx2 and COL1 were upregulated in the Ad-hBD3 and Ad-hBD3+LPS groups compared with those in the empty vector (NC) group (Fig. 2c, d). There were no significant differences between the Ad-hBD3 and Ad-hBD3+LPS groups.

### Role of the p38 MAPK pathway in the osteogenic differentiation of the hPDLCs transfected with Ad-hBD3

**Fig. 3:**
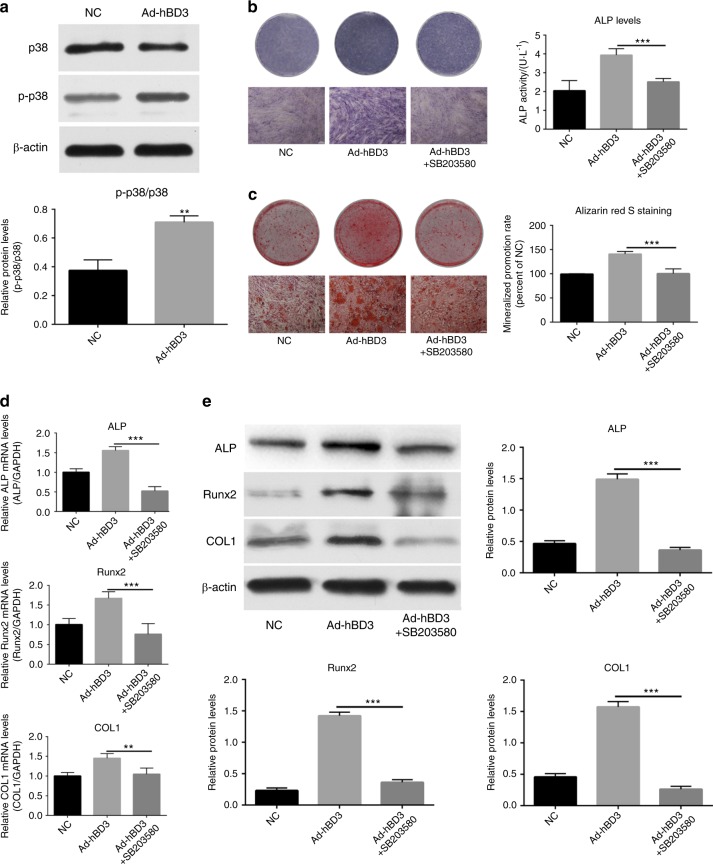
The role of the p38 MAPK pathway in the osteogenic differentiation of the hPDLCs after Ad-hBD3 transfection in an inflammatory environment. hPDLCs were transfected with Ad-hBD3 or NC (Ad-GFP) and all were treated with *E. coli* LPS (1 μg·mL^−1^). **a** p38 and p-p38 expression in the hPDLCs on day 3, **b** ALP staining and ALP activity on day 7, **c** alizarin red S staining of the mineralized nodules on day 21. **d**, **e** Bone-related gene and protein expression after adding SB203580 (**P < 0.01, ***P < 0.001)

In this experiment, all groups were assessed in the inflammatory microenvironment created with *E. coli* LPS. The relative expression of phosphorylated (p)-p38, a specific protein in the p38 MAPK pathway, was higher in the Ad-hBD3 group (Fig. 3a). The ALP activity and mineralization levels of the hPDLCs were higher in the Ad-hBD3 group than they were in the NC group. However, after adding the p38 MAPK pathway inhibitor SB203580, the promotion effect was significantly suppressed (Fig. 3b, c). Similarly, the expression of ALP, Runx2 and COL1 in the Ad-hBD3 group decreased significantly at both the mRNA (Fig. 3d) and protein levels (Fig. 3e) after SB203580 was added.

### Experimental periodontitis and rPDLC transplantation

**Fig. 4:**
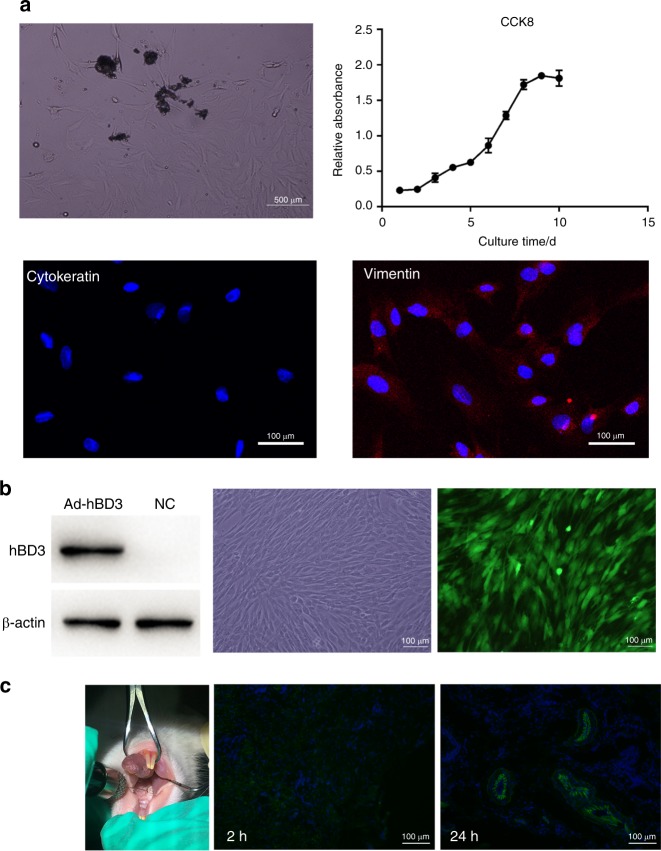
Transplantation of rPDLCs modified by Ad-hBD3. **a** Primary culture and identification of the rPDLCs, **b** fluorescence images and the hBD3 protein levels of the rPDLCs after transfection, and **c** cell injection and immunofluorescence images of the gingival tissues

rPDLCs were isolated from the PDL tissues of Sprague-Dawley (SD) rats (Fig. 4a). The immunofluorescence results showed that the cultured cells expressed vimentin (red) but not cytokeratin, which indicates that they were derived from mesoderm. The three cell growth phases, lag, log (exponential) and plateau, were observed on the cell growth curve generated from the Cell Counting Kit-8 (CCK-8) measurements. After transfection with Ad-hBD3, the rPDLCs successfully expressed hBD3 (Fig. 4b).

After ligation of the bilateral maxillary second molars, rat periodontitis models were established. rPDLCs transfected with hBD3 were transplanted into the palatal gingival tissues near the ligatured molars at mesial, middle and distal sites. Frozen sections of the injected gingival tissues were observed by confocal fluorescence after 2 h and 24 h. The images of green fluorescent protein (GFP) carried by the Ad-hBD3-transfected rPDLCs indicated that the rPDLCs had been successfully transplanted into periodontal tissues (Fig. 4c).

### Effects of the hBD3 gene-transfected rPDLCs on bone repair in the SD rats with periodontitis

**Fig. 5:**
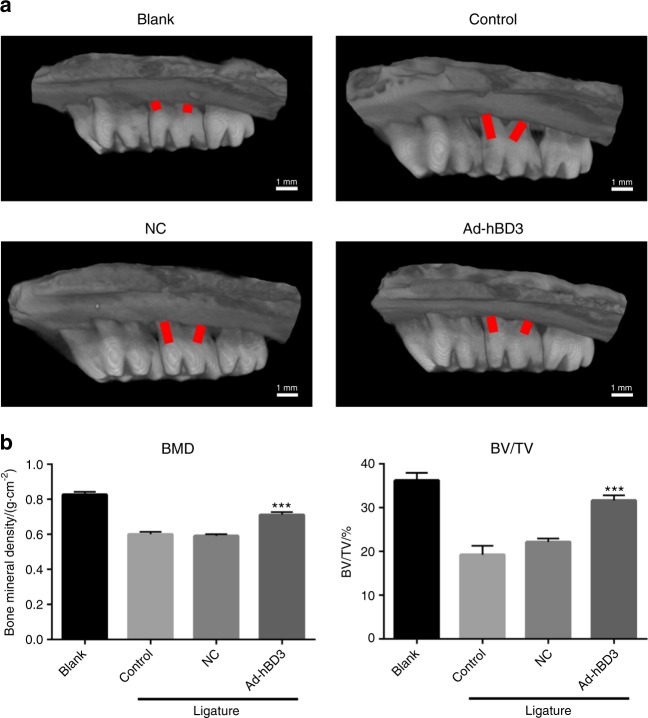
Effects of Ad-hBD3-transfected rPDLC transplantation on alveolar bone repair in the SD rats with periodontitis. **a** Three-dimensional reconstruction images, **b** bone mineral density (BMD) and the ratio of bone volume to tissue volume (BT/TV). (***P < 0.01, where triple asterisks (***) indicate a significant difference compared with that of the control group)

Two weeks after rPDLC transplantation, the bilateral maxillary bone was sampled for analysis by micro-computed tomographic (micro-CT) scanning. The 3D images showed that the alveolar bone loss around the ligatured molars of the control group was much more obvious than that of the blank group, which means that the periodontitis models were successfully created. In addition, the Ad-hBD3 group presented less bone resorption, a higher bone mineral density and a higher bone volume ratio than presented by the NC group (Fig. 5). These results indicated that the transplantation of the hBD3 gene-modified rPDLCs ameliorated bone loss and potentially promoted periodontal repair in the SD rats with periodontitis.

### Effects of hBD3 gene-transfected rPDLCs on periodontal tissue destruction in the SD rats with periodontitis

**Fig. 6:**
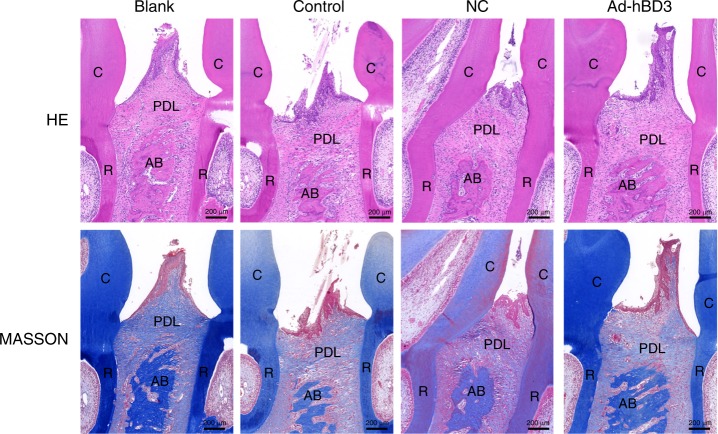
H&E and Masson’s trichrome staining of the ligatured areas. H&E and Masson’s trichrome staining were conducted to observe the morphological changes of the periodontal tissues, including gingival tissues, alveolar bone, and the periodontal ligament (original magnification: ×50; scale bar 200 μm; C, crown; R, root; AB, alveolar bone; PDL, periodontal ligament)

Haematoxylin and eosin (H&E) and Masson’s trichrome staining results showed that, in the control group, the alveolar bone around the second molars was obviously absorbed, and hyperplasia of gingival epithelial spikes and a thickened stratum spinosum were also observed, as was inflammatory cell infiltration and collagen fibre destruction. In the Ad-hBD3 group, in which the rPDLCs were transfected with the hBD3 gene, the morphology of the alveolar bone in the ligation area appeared higher and thicker compared with that of the control and NC groups. We also observed fewer inflammatory cells and lower epithelial hyperplasic spikes in the gingival epithelium, and the collagen fibres were arranged in a more orderly fashion in the Ad-hBD3 group than they were in the control group (Fig. 6).

### Effects of hBD3 gene-transfected rPDLCs on the p-p38 expression in the SD rats with periodontitis

**Fig. 7:**
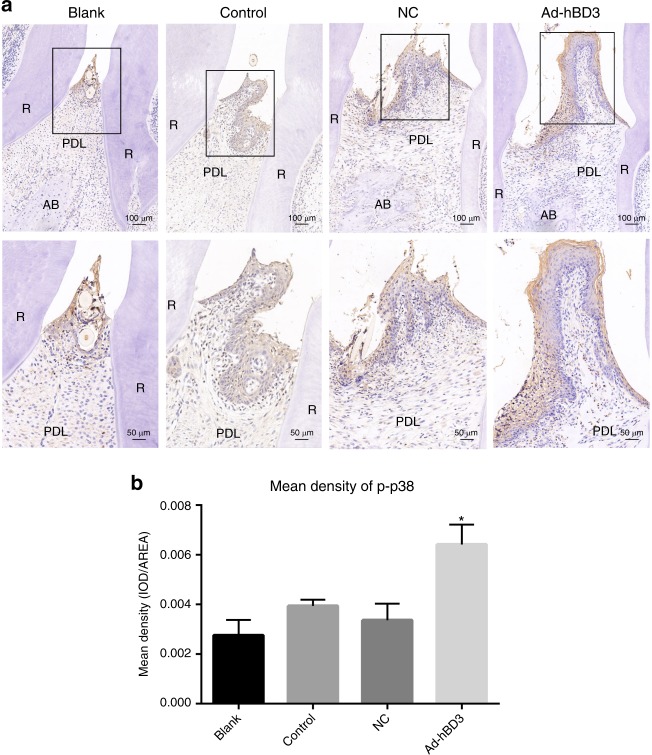
p-p38 staining and mean density (*P < 0.05, where asterisk (*) indicates a significant difference compared with that of the control group). **a** Positive expression of p-p38 in the different groups, **b** mean density of p-p38 expression in the different groups (original magnification of **a**: ×100 and ×200; scale bar 100 μm and 50 μm; R, root; AB, alveolar bone; PDL, periodontal ligament)

An immunohistochemistry assay of p-p38 was conducted to evaluate whether the p38 MAPK pathway was expressed in the rPDLCs with hBD3 gene modification applied in vivo. We found that p-p38 was expressed mostly in gingival epithelial tissues. The blank and control groups showed low levels of p-p38 expression, while the Ad-hBD3 group exhibited the highest p-p38 expression levels (Fig. 7a). The mean density of p-p38 in the Ad-hBD3 group was significantly higher than that in the control group (Fig. 7b).

## Discussion

Inflammation-induced bone loss has recently become a research hotspot due to the profound effects of inflammatory responses on local and systematic bone metabolism.^1^ Periodontitis is considered to be one of the diseases with inflammatory responses and destruction of periodontal tissues, including evident alveolar bone loss.^32,33^ In our study, 1 μg·mL^−1^ *E. coli* LPS was chosen to create the inflammatory microenvironment, because LPS was proven in previous studies to induce an hPDLC inflammatory response.^34,35^

hBD3 is known as a kind of cationic antimicrobial peptide generally considered to have excellent antibacterial activity and certain immunomodulatory functions.^18,36^ In this study, we evaluated its effect on the osteogenesis of hPDLCs. hPDLCs with overexpressed hBD3 were constructed have more sustainable and stable expression of hBD3. Gene delivery technologies have become an emerging approach in biomedical fields.^37,38^ High transduction efficiency and low insertional mutagenesis have rendered adenoviral vectors as attractive gene delivery vehicles.^39^ Moreover, adenoviral vectors exhibit strong and sufficient effects.^40,41^ In our study, hPDLCs transfected with adenovirus containing the hBD3 gene were found to express the hBD3 protein for at least 7 days, a finding consistent with the results of a previous study.^42^ Acting as a vital component of the periodontium, hPDLCs participate in periodontal tissue regeneration and are widely used in tissue engineering because of their characteristic ability to produce collagen and bone-associated proteins, such as OCN and osteopontin.^6,18,43^ In this study, in addition to remarkable ALP activity and impressive mineralized nodules, hBD3 gene-modified hPDLCs presented with significant expression of osteogenic proteins; all of these results indicated that hBD3 promoted osteogenic differentiation of the PDLCs.

Primary culture rPDLCs modified by hBD3 gene transfection were transplanted into the rat periodontitis models. The results from the micro-CT and histology assays showed obvious bone repair and alleviated inflammatory response in the Ad-hBD3 group. In vitro, we proved that hBD3 gene transfection promoted the expression of osteogenic indicators and osteogenic differentiation of the PDLCs. After transplantation, the hBD3 gene-modified rPDLCs might have had better osteogenesis ability to enhance osteogenesis during the bone remodelling process. The specific mechanism might involve the regulation of angiogenesis and the recruitment of stem cells,^30^ or the inhibited osteoclast formation and bacterial activity,^44^ as indicated by previous studies with other antimicrobial peptides.^45^ We have demonstrated that hBD3 enhanced the osteogenesis of the PDLCs and p-p38 expression in vitro. After adding p38 MAPK pathway inhibitor SB203580, the promotion effect was significantly suppressed, which means that the process might be regulated by the p38 MAPK pathway in vitro. Similarly, we also detected higher expression levels of p-p38 in the hBD3 gene-modified rPDLC transplantation group than we found in the control group in vivo. However, in vivo environments are much more complicated owing to the multiple signalling pathways involved, and the MAPK pathway may play only a potential role in the process of bone repair in vivo. Further research concerning the detection of downstream targets of the MAPK pathway is also needed to identify the specific mechanisms. Furthermore, it is important for long-term tracing and the observation of transplanted cells in vivo. More research is needed to further observe the localization of transplanted PDLCs and the expression of related bone markers. The inherent antibiotic and immunomodulatory characteristics of hBD3 may have affected the osteogenic differentiation of hBD3 gene-modified PDLCs. More research is needed with a control group consisting another antibiotic agent to confirm the mechanism.

In conclusion, our study evaluated the osteogenic effect of hBD3 gene-modified hPDLCs in vitro and in vivo. The hBD3 gene-modified hPDLCs showed greater osteogenic ability in vitro through the p38 MAPK pathway. Furthermore, hBD3 was able to alleviate the inflammatory destruction of periodontitis along with the promotion of bone repair in vivo. hBD3 may play multiple roles in the treatment of periodontitis, and hBD3 gene-modified hPDLC transplantation may be a potential approach for the treatment of periodontitis.

## Materials and methods

### hPDLC culture

hPDLCs from healthy donors were obtained from ScienCell (ScienCell Research Laboratories, San Diego, CA, USA). The cells were cultured in Dulbecco’s modified Eagle’s medium (Gibco, Grand Island, NY, USA) with 1% penicillin/streptomycin (Gibco, Grand Island, NY, USA) and 10% foetal bovine serum (ScienCell Research Laboratories, San Diego, CA, USA) at 37 °C in 5% CO_2_. Every other day, the medium was refreshed, and the cells between passages 2 and 6 were used. For the osteogenic differentiation test, osteogenic differentiation medium (growth medium supplemented with 50 μg·mL^−1^ ascorbic acid (Sigma-Aldrich, St. Louis, MO, USA), 10 mmol·L^−1^ β-glycerophosphate (Sigma-Aldrich, St. Louis, MO, USA) and 100 nmol·L^−1^ dexamethasone (Sigma-Aldrich, St. Louis, MO, USA) was used to replace the original growth medium. The culture medium was refreshed every 2 days.

### Adenoviral vectors and gene transfection

The recombinant adenovirus, which carried the GFP (NC, Ad-GFP) or hBD3 gene (Ad-hBD3), was purchased from GenePharma (GenePharma, Shanghai, China). The hPDLCs (1.0 × 10^5^ cells per well) were seeded in 6-well plates. After reaching 80% confluence, the cells were transfected with Ad-GFP (NC) or Ad-hBD3 at different MOI values, ranging from 100 to 200. GFP was used as the reporter molecule to assess the transfection efficiency of Ad-hBD3, and the infection efficiency was determined by flow cytometry. Ad-hBD3-hPDLCs were assayed for hBD3 expression by WB.

### Quantitative real-time PCR

The mRNA levels of osteogenesis-related genes were determined by qRT-PCR. Six-well plates with growth medium were chosen to seed hPDLCs (1 × 10^5^ cells per well). Transfection of Ad-GFP (NC) and Ad-hBD3 was conducted when the cell fusion rate reached 80%. On day 3, after hBD3 expression was detected, *E. coli* LPS (1 μg·mL^−1^) was added to the medium and incubated for 24 h to simulate an inflammatory microenvironment.^46,47^ TRIzol reagent (Tiangen, Beijing, China) was used to extract total RNA from the cells. The reverse transcription of total RNA to cDNA was performed by using the PrimeScript RT Reagent Kit (TaKaRa, Otsu, Japan). The sequences of the primers for qRT-PCR are shown in Supplementary Material. Each gene cycle threshold (ct) was normalized based on the ct of glyceraldehyde 3-phosphate dehydrogenase, which was determined simultaneously on the same plate, and then calculated by the comparative 2^−ΔΔCt^ method. All of the samples were run in triplicate.

### WB analysis

The expression levels of hBD3, osteogenesis-related, p38, and p-p38 proteins were measured by WB assay. Cells were lysed in RIPA buffer (Beyotime, Shanghai, China). The total proteins were denatured by boiling for 5 min, resolved by 10% gradient sodium dodecyl sulfate-polyacrylamide gel electrophoresis and then transferred onto polyvinylidene difluoride membranes (Millipore, Bedford, MA, USA). Five percent skim milk powder was used for blocking; 2 h later, the membranes were incubated overnight with primary antibodies, anti-hBD3 (ab19270), anti-ALP (ab83259)), anti-Runx2 (ab23981), anti-COL1 (ab96723) (Abcam, Cambridge, UK), anti-p38 (#8690 S) and anti-p-p38 (#4511 S) (CST, MA, USA), with anti-β-actin (BS6008M) (Bioworld, MN, USA) as the housekeeping gene, at 4 °C, and then anti-rabbit or anti-mouse secondary antibody was added and incubated for 1 h. A Tanon 5200 chemiluminescent imaging system (Tanon, Shanghai, China) was utilized to visualize the proteins.

### ALP activity and staining assay

An ALP assay kit (Abcam, MA, USA) was used to assess ALP activity. After transfection, hPDLCs (3.0 × 10^4^ cells per well) were seeded into 24-well plates. After overnight culture, the medium was changed into an osteogenic differentiation medium with AuNPs (45 nm, 10 μmol·L^−1^). After culturing for 7 days, the cells were rinsed twice with phosphate-buffered saline (PBS). After a series of steps following the manufacturer’s instructions, the final solution was added to the plates. The absorbance was assessed at 405 nm by a SpectraMax M3 microplate reader (Molecular Devices, Sunnyvale, CA, USA). The ALP activity level was determined, relative to that of the control group, as a percentage against a standard curve. The extent of the ALP staining was determined on the same day. The plates were rinsed twice with PBS and then fixed in 4% paraformaldehyde for 30 min. Next, the BCIP/NBT ALP Staining Kit (Beyotime Institute of Biotechnology, Shanghai, China) was used for cell staining according to the manufacturer’s instructions. The stained plates were air-dried and examined under a light microscope (Olympus IMT-2, Tokyo, Japan) and photographed with a digital camera (Canon EOS 550D, Tokyo, Japan).

### ARS staining

The cells in all groups were incubated in osteogenic medium for 3 weeks and then rinsed twice with PBS and fixed in 4% paraformaldehyde for 30 min. The cells were washed with distilled water (DW), treated with 2% ARS solution (Sigma-Aldrich, USA) for 5 min and then washed 3–5 times with DW to remove unbound ARS. The stained plates were air-dried and examined under a light microscope (Olympus IMT-2, Tokyo, Japan) and photographed with a digital camera (Canon EOS 550D, Tokyo, Japan). For the quantification of ARS staining, the cells were desorbed with 10% (w/v) cetylpyridinium chloride (Sigma-Aldrich, St. Louis, MO, USA); then the absorbance was measured at 562 nm by a SpectraMax M3 microplate reader.

### Culture and identification of rPDLCs

All animal experiments were approved by the Ethics Committee of Nanjing Stomatological Hospital, Medical School of Nanjing University. The animal experiments were conducted at the Nanjing Mergene Biotechnology Development Co., Ltd. (accreditation number: SYXK 2017-0066) according to the policies and guidelines for institutional animal care of Nanjing University, Nanjing, China. Five-week-old adult female SD rats were sacrificed, and then their six molars were extracted and placed into 0.1% collagen I solution and shaken for 2 h at 37 °C. After centrifugation, the cells were resuspended in growth medium at 37 °C in 5% CO_2_ for 3–5 days, and then the level of cell adherence was observed. The culture medium was refreshed every 2 days. After 1–2 passages, the cells were fixed with 4% paraformaldehyde for the subsequent immunofluorescence staining of anti-cytokeratin and anti-vimentin (CST, MA, USA), which were used to identify the cell origin. The cells were seeded into 96-well plates (5.0 × 10^3^ cells per well), cultured for 10 days, and then 10 μL of the reagent from CCK-8 was added to each well on each day and incubated at 37 °C in 5% CO_2_ for 4 h. The absorbance was measured with a SpectraMax M3 microplate reader (Molecular Devices, Sunnyvale, CA, USA) at a wavelength of 450 nm. Cell viability was calculated as the relative absorbance after excluding the background absorbance.

### Experimental periodontitis and rPDLC transplantation

Twenty female SD rats (5 weeks old) were randomly allocated into 4 groups (each group with 5 SD rats): a blank group (without ligature or cell transplantation), control group (with ligature alone), NC group (with ligature and cell transplantation treated with empty vector), and Ad-hBD3 group (with ligature and cell transplantation treated with Ad-hBD3). Silk threads were soaked in a bacterium solution of *P. gingivalis* for 2 h ahead, and then they were used to ligature the bilateral maxillary second molars of the SD rats. The rPDLCs subjected to different treatments were dissociated into cell suspensions with 0.9% NaCl (1 × 10^4^ cells per μL) and then injected with a 100-μL micro-syringe (Hamilton, Bonaduz, Switzerland) into the mesial, middle and distal sites of the palatal gingival tissues around the ligatured molars. The palatal side was selected for transplantation to provide the best possible visual field and operability, and the transplanted cells could migrate around the entire tooth. After 2 and 24 h, the relevant gingival tissues were collected and processed into frozen sections to confirm a successful rPDLC transplantation. rPDLC transplantations were performed once per week, and 2 weeks later, all rats were sacrificed, and samples were taken.

### Micro-CT scanning

The maxillary bone samples of the SD rats were trimmed and placed into 4% paraformaldehyde fixative solution for 24 h. The next day, the samples were collected and prepared for micro-CT scanning with a Skyscan 1176 scanner (Bruker, Karlsruhe, Germany). The scanning parameters were as follows: a rotation angle of 360°, a tube voltage of 70 kV, a tube current of 353 μA, an X-ray exposure time of 404 ms, and a scanning layer thickness of 18 μm. The data were reconstructed with the NRecon software and then imported into the CTVox and CTAn software to obtain 3D images and relative data.

### Histological analysis and immunohistochemistry assay for p-p38 detection

Two weeks after rPDLC transplantation, the SD rats were sacrificed by euthanasia. The right maxillas were collected and fixed in 4% paraformaldehyde for 48 h and then placed into 10% EDTA decalcifying solution for 1 month. Afterward, the specimens were dehydrated with a gradient series of 40%, 50%, 60%, 70%, 80%, 90%, and 95% ethanol for 12 h each at each stage, and they were finally soaked in a 95% ethanol and xylene solution mixture (1:1) for 12 h to make them transparent. After sectioning the samples to a thickness of 5 μm, they were stained with H&E and Masson’s trichrome. To conduct the immunohistochemistry assay of p-p38, the sections were stained with p-p38 antibody, and the mean density of p-p38 was measured in three different samples from the same group. A light microscope was used to observe the local histological structures.

### Statistical analysis

In our study, in vitro experiments were repeated at least three times, and in vivo experiments included at least three samples from each group to reduce errors and support the statistical analysis. Statistical calculations were performed with the SPSS 23 statistical software (SPSS, Chicago, IL, USA). Depending on the context, significant differences were determined using Student’s *t* test or one-way analysis of variance (ANOVA) followed by Bonferroni test. Significance was determined on the basis of the independent sample *t* test or ANOVA and was indicated with *P* values < 0.05.

## Supplementary information

Supplementary Material
