# Supplementary material for: Human β-defensin 3 gene modification promotes the osteogenic differentiation of human periodontal ligament cells and bone repair in periodontitis
Source: Int J Oral Sci. 2020 Apr 29;12:13. doi: 10.1038/s41368-020-0078-6 (PMC7190824; doi:10.1038/s41368-020-0078-6)
Supplement: Supplementary file 1 — Supplementary Material [file 41368_2020_78_MOESM1_ESM.docx]

**Supplementary Material**

Table 1. Primer sequences.

The sequences of the primers for qRT-PCR

| Primer name | Forward primer sequence (5’-3’) | Reverse primer sequence (5’-3’) |
| --- | --- | --- |
| ALP | CCGTGGCAACTCTATCTTTGG | GCCATACAGGATGGCAGTGA |
| Runx2 | AACCCACGAATGCACTATCCA | CGGACATACCGAGGGACATG |
| COL1 | CTGCAAGAACAGCATTGCAT | GGCGTGATGGCTTATTTGTT |
| GAPDH | GGCGTGATGGCTTATTTGTT | GGCGTGATGGCTTATTTGTT |
